# 

**Published:** 1887-05-21

**Authors:** 


### The Hospital.

An Institution, Family, and Parochial Journal of Hospitals, Asylums, and all Agencies for the Care of the Sick, Criticism and News.

SATURDAY, MAY 21st, 1887.

### Notice to Advertisers.

*To insure prompt insertion, all Advertisements for* The Hospital *must be sent in direct to the Advertising Agent, Mr. A. P. Watt, 2, Paternoster Square, E.C.*

### The Literary Prize.

A prize of **Two Guineas** will be given every month for the best story or incident based upon hospital, or asylum, or student life, or any incident connected with the professions of medicine or nursing.

*** The Children's Corner and Amusements with Profit will be found on page vi of this week's issue.

### QUESTIONS AND ANSWERS.

All questions should be addressed to Mr. Howard J. Collins, Secretary, The Hospitals Association, Norfolk House, Norfolk Street, Strand, W.C.

### "MATRONS AND SISTERS AT FIFTY."

"Kindly tell me for how many years is a matron, or sister, required to pay into the National Pension Fund before she could receive a pension. There are many matrons who are close on fifty years of age; consequently, cannot expect to bear the strain of hospital work much longer. An answer in your next issue will much oblige."

*** The question raised will have to be determined later on. It is usual in Funds of this kind to meet these cases, by allowing the applicant to pay up back years in a lump sum, and so to join the fund. Actuaries have, however, the strongest objection to this method, and prefer entire exclusion, or a very high rate for the few years during which the applicant will have to pay. In any case, whatever special exceptions may be made at the outset, no one will be accepted ultimately, we imagine, after attaining a maximum age of say forty years.—[Ed. T. H.]

### THE QUILTER PRIZE FORM.

Mr. H. Lawford Jones writes from the Bristol Hospital for Sick Children:—"What is the difference between the following questions, asked on the Quilter Prize Form—(1) Average number of patients resident throughout the year; (2) Number of beds constantly occupied? Also, you have the *total* cost per occupied bed. What is the *average* cost?"

*** The average number of patients resident throughout the year includes those who remain in the hospital on the 31st December, or whatever date the books are closed, whilst the number of beds constantly occupied represents the number of beds which contained one patient on each and every day throughout the whole year. The average cost per occupied bed is the average cost as calculated by the hospital authorities on a three years' average.

### THE IN- AND OUT-PATIENTS' HOSPITAL DIARY.

This Diary is so arranged as to make some portion of it appear every week, and the whole in each monthly part. It has been verv difficult to compile, and corrections will at all times be gratefully received. It has been suggested that similar information about the larger Provincial and Scotch Hospitals would be useful to many readers. We should be glad to hear if this is felt to be the case.

### ORTHOPÆDIC CASES.

| Hospitals and Terms of Admission. | Physicians, Surgeons, and Medical Officers, with Days and Hours of Attendance. |
|---|---|
| City Orthopædic Hospital, 27, Hatton Garden, E.C. Sec., Mr. E. Dereuth. Admission free | *Surgeons*, Mr. E. J. Chance and House-Surgeon. M. Tu. Th. F. 2.30. New cases Tu. F. 2.30 |
| National Orthopædic Hospital for the Deformed, 234, Gt. Portland St., W. Sec., Mr. H. Canning. Free to poor; others by letter or payment | *In-Physician*, Dr. C. Beevor. *In- and Out-Surgeons*, Messrs. F. R. Fisher, M. Th. 2; W. J. Roeckel, Tu. F. 2; E. M. Little, W. 1 |
| Royal Orthopædic Hospital, 297, Oxford Street, W. Sec., Mr. B. Maskell. By Governor's letter | *Surgeons*, Messrs. B. E. Brodhurst, H. A. Reeves, C. Read, W. E. Balkwill, H. F. Baker. Daily, 1 |
| St. Bartholomew's Hospital, Smithfield, E.C. | *Surgeon*, Mr. Walsham, M. 2.30 |
| St. George's Hospital, Hyde Park Corner, S.W. | W. 2. *See* General Hospitals |

### PARALYSIS, EPILEPSY, AND DISEASES OF THE NERVOUS SYSTEM GENERALLY.

| | |
|---|---|
| Hospital for Epilepsy and Paralysis, and other Diseases of the Nervous System, 32, Portland Terrace, Regent's Park Road, N.W. Sec., Mr. Howgrave Graham. By contributor's letter, or payment according to means, but free to necessitous poor | *In-Physicians*, Drs. Hughes Bennett, M.; G. Ogilvie, Tu.; J. Althaus, Th. Hour, 2. *Out-Physicians*, Drs. H. Bennett, M.; G. Ogilvie, Tu.; W. H. Syers, W.; J. Althaus, Th.; Laycock, F. Hour, 2. *In- and Out-Surgeons*, Messrs. J. Bloxam, A. P. Gould, when required |
| National Hospital for the Paralysed and Epileptic, Queen Square, Bloomsbury, W.C. Sec. and Director, Mr. B. B. Rawlings. Most of the beds are reserved free to the necessitous poor; the others are for patients who contribute a Guinea a week | *In-Physicians*, Drs. C. B. Radcliffe, M.; J. S. Ramskill, Tu.; T. Buzzard, W.; J. H. Jackson, F. Hour, 2.30. *In-Surgeons*, Messrs. W. Adams, M. 3; V. Horsley, F. 2.30. *Out-Physicians*, Drs. W. R. Gowers, M.; H. C. Bastian, Tu.; J. A. Ormerod, Tu. W.; T. Buzzard, W.; D. Ferrier, F.; C. E. Beevor, M. F. Hour, 2.30 |
| National Hospital for Diseases of the Heart and Paralysis, 32, Soho Sq., W. Sec., Capt. F. Handley. In-patients require recommendation of Life Governor. | *In-Physicians*, Drs. A. Duncan, J. Murray, Stretch Dowse, M. W. Th. *In-Surgeon*, Mr. Graham Bennett, Th. *Out-Physicians*, Drs. R. Verley, J. Murray, Stretch Dowse, Tu. Th. F. 3; M. W. 3 and 8. *Out-Surgeon*, Mr. G. Bennett, Th. 3 |
| West End Hospital for Diseases of the Nervous System, 73, Welbeck St., W. Hon. Sec., Mr. H. A. Dowell. Children only as in-patients. Out-patients by letter or payment. | *In- and Out-Physicians*, Drs. H. Tibbits, M. Th. 1.30; Stretch-Dowse, Tu. W. 1.30; S. H. Armitage, W. Tu. F. 6. *In- and Out-Surgeon*, Mr. Benson |

### DISEASES OF THE SKIN.

| | |
|---|---|
| Charing Cross Hospital, West Strand, W.C. | *Physician*, Dr. Sangster, M. 2 |
| Guy's Hospital, St. Thomas Street, S.E. | *Physicians*, Drs. Hale-White, Pye-Smith, Tu. 12 |
| Hospital for Diseases of the Skin, 52, Stamford St., S.E. Sec., Mr. S. Hayman. Free to the necessitous; also by payment | *Physician*, Dr. F. J. Payne. *Surgeons*, Messrs. J. Hutchinson, Waren Tay, and Wyndham Cottle. (Out-patients seen M. W. 3 Tu. Th. F. 2) |
| King's College Hospital, Portugal Street, W.C. | *Physician*, Dr. Duffin, F. 1 |
| London Hospital, Whitechapel Road, E. | *Physician*, Dr. S. Mackenzie, Th. 9 |
| Middlesex Hospital, Berners Street, W. | *Physician*, Dr. R. Liveing, Tu. 4 |
| North-West London Hosp., 18, Kentish Town Rd., N.W. | *Physician*, Dr. J. Stowers, Tu. 1.30 |
| Paddington Green Children's Hospital, Paddington | *Physician*, Dr. T. Colcott Fox, S. 9.15 |
| St. Bartholomew's Hospital, Smithfield, E.C. | *Surgeon*, Mr. H. Cripps, F. 1.30 |

### DISEASES OF THE SKIN.—*Continued.*

| Hospitals and Terms of Admission. | Physicians, Surgeons, and Medical Officers, with Days and Hours of Attendance. |
|---|---|
| St. George's Hospital, Hyde Park Corner, S.W. | *Physician*, Dr. Cavafy, W. 1.30 |
| St. John's Hospital for Diseases of the Skin, 45, Leicester Sq., W.C. Branch, Markham Square, Chelsea, S.W. Sec., Mr. St. Vincent Mercier. In-patients by letter; out-patients free or by small payment | *Physicians*, Drs. Bourns, Campbell, Dow, Harries, Robinson. Daily 2 and 7. Markham Sq. Branch, M. 10 and Th. 10 and 7. *Surgeon*, Mr. J. Milton, Tu. |
| St. Mary's Hospital, Cambridge Place, Paddington, W. | *Surgeon*, Mr. Morris, M. Th. 9.30 |
| St. Thomas's Hospital, Westminster Bridge Road, S.E. | *Physician*, Dr. Payne, W. 1.30 |
| University College Hospital, Gower Street, W.C. | *Physician*, Dr. Crocker, W. 1.30, S. 9.15 |
| Westminster Hospital, Broad Sanctuary, S.W. | *Physician*, Dr. T. C. Fox, W. 1.30 |

### STONE AND OTHER DISEASES OF URINARY ORGANS.

| | |
|---|---|
| London Hospital, Whitechapel Road, E. | Daily, 1 30. *See* General Hospitals |
| St. Peter's Hospital for Stone and Urinary Diseases, Henrietta Street, W.C. Sec., Mr. W. E. Scott. Admission free. Only women and children are seen on Fridays. Operation day, Wednesday | *In-Surgeons*, Messrs. W. J. Coulson, F. R. Heycock. *Out-Surgeons*, Messrs. E. H. Fenwick, M. 2, S. 4 to 7; F. S. Edwards, M. 5 to 7, Th. 2; Heycock, Tu. 2, W. 5 to 7, F. 2 |

### DISEASES AND IRREGULARITIES OF THE TEETH.

| | |
|---|---|
| Belgrave Hosp. for Children, 77 & 79 Gloucester St., Pimlico | *Surgeon*, Mr. G. W. Payne, W. 9 |
| Charing Cross Hospital, West Strand, W C. | *Surgeon*, Mr. Fairbank, M. W. F. 9.0 |
| Dental Hospital of London, Leicester Sq., W.C. Sec., Mr. J. F. Pink. Poor free. For special operation a Governor's letter is required | *Surgeons*, Messrs. S. Bennett, Canton, Gregson, Hepburn, Hern, Matheson, Parkinson, Read, Rogers, Truman. Underwood, Woodhouse. Daily, 9 to 11 |
| Evelina Hosp. for Sick Children, Southwark Brdg. Rd. | *Surgeon*, Mr. R. Denison Pedley, Tu. 9 |
| German Hospital, Dalston lane, Dalston, E. | *Surgeons*, Messrs. A. P. Reboul, C. West, Th. 10 to 11 |
| Great Northern Central Hospital, Caledonian Rd, N. | *Surgeon*, Mr. E. Keen, W. 1.30 |
| Guy's Hospital, St. Thomas Street, S.E. | *Surgeons*, Messrs. Moon, Pedley, Tu. Th. F. 12.30 |
| Hospital for Consumption, Brompton, S.W. | *Surgeon*, Mr. C. J. Noble, W., and when wanted |
| Hospital for Sick Children, Gt. Ormond Street, W.C. | *Surgeon*, Mr. Cartwright, W. 9 |
| King's College Hospital, Portugal Street, W.C. | *Surgeon*, Mr. Cartwright, Tu. F. 10 |
| London Hospital, Whitechapel Road, E. | *Surgeon*, Mr. A. W. Barrett, Tu. 9 |
| London Temperance Hosp., Hampstead Road, N.W. | *Surgeon*, Mr. Adolphus Alexander, M. 2 |
| Metropolitan Free Hospital, Gray's Inn Road, W.C. | *Surgeon*, Mr. G. Curnock, Tu. Th. S. 9 |
| Middlesex Hospital, Berners Street, W. | *Surgeons*, Messrs. Bennett, M. 9.30; W. 9; Hern, W. 9, F. 9.30 |
| National Dental Hospital, 149, Gt. Portland Street, W. Sec., Mr. A. G. Klugh. The poor, and all urgent cases admitted free; others by Subscriber's letter | *Surgeons*, Messrs. H. Weiss, W. Weiss, M.; A. Smith, G. Bradshaw, Tu.; G. A. Williams, M. Davis, W.; A. F. Canton, H. G. Read, Th.; T. Gaddes, W. S. Thomson, F.; H. Rose, W. R. Humby, S. Hours 9 to 11 |
| National Orthopædic Hospital, 234, Gt. Portland st, W. | *Surgeon*, Mr. H. Harris |

### THE CHILDREN'S CORNER.

**Third Quarterly Prize Competition.**
Began 2nd Apr., 1887 ; ends 25th June, 1887.

### Puzzles—Eighth Week.

No. 31. CONUNDRUM. What articles of an old lady's attire most resemble solitary recluses ?

No. 32. ENIGMA.

Three sisters wander o'er the earth,
 Three sisters fair and good,
The second woke at Nature's birth
 In Eden's solitude.
The eldest's life, it ne'er began
 Till reverence fill'd the soul of man,
The youngest, lovely as a dream,
 In life—in death reigneth supreme.
Tell me who are these sisters three,
Who wander forth o'er land and sea.

No. 33. BIRDS' NAMES ENIGMATICALLY EXPRESSED. Quick and rapid ; a monarch and an angler ; timber used for masts and a noisy disturbance ; everything, a racquet and a county in Scotland.

```
    *
  • * •
* * * * *
  • * •
    *
```

No. 34. DOUBLE MESOSTICH. A vowel; a river in England ; what we should all strive to do ; a river in Scotland ; the beginning of love.

### Letters—Eighth Week.

The Letter-subject for next week will be "The Garden."

### Results of Sixth Week—Puzzles.

No. 24. BURIED NAMES OF BIRDS. Jay, raven, martin, lark.

No. 25. CHARADE. Ear-nest = earnest.

No. 26. CONUNDRUM. An anchor.

| | | | |
|---|---|---|---|
| 1 | 2 | 3 | 4 |
| 5 | 6 | 7 | 8 |
| 9 | 10 | 11 | 12 |
| 13 | 14 | 15 | 16 |

No. 27. SQUARE PUZZLE. The boy first enters No. 2. The conditions say that he is to *enter each square*, and as he was already in the first, it is evident that he has still to enter it. He accordingly leaves No. 2 and enters No. 1. His course is then simple enough, viz., 5, 6, 10, 9, 13, 14, 15, 11, 7, 3, 4, 8, 12, and 16. Several other routes are possible. The catch is at the start.

*All right*—Gom, Nordisa ; *Three right*—A. C. K., Agatha, Alsie, Bohemian Girl, Dot, Ellie, R. Epton, Joe, A. G. S. McCulloch, Mikado, Scrooge, Skye, Sutton, Tim, Umslopogaas ; *Two right*—Peeler, Etta ; *One right*—Ada.

### Letters.

The letters about "Music" were, on the whole, scarcely up to the average, though two or three were exceptions. Mikado is exceedingly musical, Beethoven and Mendelssohn are, she tells me, her favourite composers. Ada writes so philosophically that I can hardly fancy the composition is that of a little girl of twelve ; is it so ? Peeler pens a clever letter about The Messiah. Etta has just begun to learn, and Ellie says that she is shortly to do so. Skye, whose tastes lie in the direction of the limner's art, does not care for music. I hope next week to receive some very interesting letters, for the garden is a subject on which everyone ought to be able to write.

One mark each:—Bohemian Girl, Ellie, Mikado, Peeler, Skye, Ada, Etta.

### Notices to Correspondents.

SKYE.—Many thanks for the pretty photo you have so kindly sent me.

SUTTON.—Answers must be received by Thursday. In future, they will be disqualified if not received in time.

Answers must be addressed to the "PUZZLE EDITOR," THE HOSPITAL, Norfolk House, Norfolk Street, Strand, W.C., not later than Thursday next. The "Children's Corner" Coupon (see advertisement pages) must be enclosed.

### TO ADVERTISERS.

**Cheap Advertisements** of the "Wanted" class, including those for situations, of Twenty-four Words or under, are charged 1*s.* an insertion, or three insertions for 2*s.* 6*d.*, but must be paid for in advance. Each additional line of Eight Words is 4*d.*

**Employers requiring assistants** are charged for one insertion of Twenty-four Words or under, 2*s.* 6*d.*, or 6*s.* for three insertions. Each additional line of Eight Words is 1*s.*

### AMUSEMENTS WITH PROFIT.

### PRIZE COMPETITIONS.

**Quarterly Prize Competition.**
Began 23rd April ; ends 16th July.

**A Prize of Three Guineas**

will be awarded to the Competitor who shall have sent in the largest number of correct or best answers. No one will be permitted to win the Three Guinea Prize twice in any one year, but we shall award annually an additional

**Prize of Five Guineas**

to the competitor who shall have sent in the largest number of correct or best answers during the twelve months.

### No. 9. Double Acrostic.

Here the sick young are nurs'd with care,
The lights will show the name and where.
* * * * * *
What ev'ry barrister would hold ;
Purveyor of fruit-salt far extoll'd ;
 Nor this nor a borrower be ;
These are by ev'ry lawyer claim'd ;
A theatre from the Adams nam'd ;
 'Gainst quoted words, if question'd be ;
Once Europe's terror—some still hate ;
To Afric's tongues this doth relate ;
 From this, some M.P.'s would be free ;
Thus was Abraham's wife first nam'd ;
This gem is sought in fish'ries fam'd ;
 Troy's woes this poem tells at length ;
The *Erebus* this calls to mind ;
To win back these, France long hath pin'd,
 'Tis the emblem of British strength.

### No. 10. Distinguished Doctors.

Name the twelve greatest doctors, English, Scotch, or Irish. Only doctors of medicine are, of course, referred to. No names of living medical men are to be given. We shall tabulate from the lists sent in, the names in order of the number of the votes they receive, and the twelve who stand highest will form the accepted list. We shall credit as "best answers" each list which has four (or more) of the names appearing in the accepted list. (No answer can be considered which does not arrive by the first post on Saturday.)

### Answers.

No. 5. DOUBLE ACROSTIC.

| | | |
|---|---|---|
| A | nne Aske | W |
| M | achiavell | I |
| U | trech | T |
| S | out | H |
| E | astchea | P |
| M | assinge | R |
| E | ch | O |
| N | icolaief | F |
| T | ivol | I |
| S | outhco | T |

As "Sidney Smith" will exactly meet the conditions of the fourth light, we accept it.

Correct answers have been received from Ellice, Gip, The Man of the Sea, Dawnlover, May, A. M. E., Gretta, Mater, Aralc, Broadwater, K. M., Letheàdor, Canice, Ostrich, Robin, Grip, Boss, Tabs, Isis, Mrs. Bedingfeld, Mr. J. High.

No. 6. MATHEMATICAL QUESTION.

Let $x$ = price of horse.

Then $x + \frac{x^2}{100} = 144$

$\therefore x^2 + 100x = 14.400$

or $x^2 + 100x - 14,400 = 0$

$\therefore (x + 180)(x - 80) = 0$

$x = £80$

Correct answers have been received from Perseverance, Ellice, Gip, The Man of the Sea, Dawnlover, May, A. M. E., Gretta, Mater, Aralc, Broadwater, K. M., Peeler, Letheàdor, Canice, Ostrich, Robin, Grip, Boss, Tabs, Hexameter.

"HELP GUY'S."—The following sums are acknowledged, A. M. E., 2*s.* 6*d.*, Mater, 1*s.*, amount previously acknowledged ; 4*s.* ; total, 7*s.* 6*d.*

### Notices to Correspondents.

In future we must disqualify solvers who habitually exceed the date fixed for sending in, or who do not address their letters according to instructions given below.

Answers must be addressed to THE PRIZE EDITOR, AMUSEMENTS WITH PROFIT, THE HOSPITAL, Norfolk House, Norfolk Street, Strand, W.C., not later than the *first* post on Saturday next. The full name and address must in all cases be given, but a *nom de plume* may be added for publication. Only one side of the paper should be written on. The "Amusements with Profit" Coupon (see advertisement pages) must be enclosed with replies.

